# COVID-19 vaccine delivery: an opportunity to set up systems for the future

**DOI:** 10.12688/gatesopenres.13210.1

**Published:** 2020-12-18

**Authors:** Rebecca Weintraub, Stanley Plotkin, Margaret Liu, Jerome Kim, Natalie Garcon, David Bell, Dan Storisteanu, Toby Norman, Eliah Aronoff-Spencer

**Affiliations:** 1 Ariadne Labs, Harvard T.H. Chan School of Public Health and Brigham and Women’s Hospital Boston, Boston, MA, 02215, USA; 2 Harvard Medical School, Cambridge, MA, USA; 3 University of Pennsylvania School of Medicine, Philadelphia, PA, USA; 4 ProTherImmune, Lafayette, CA, USA; 5 International Vaccine Institute, Seoul, Seoul, South Korea; 6 Bioaster Technology Research Institute, Lyon, France; 7 Independent Researcher, Seattle, Washington, USA; 8 University of Cambridge, Cambridge, UK; 9 Simprints, Cambridge, UK; 10 School of Medicine, Division of Infectious Diseases and Global Public Health, University of California San Diego, La Jolla, Ca, 92093, USA

**Keywords:** vaccine deliver, biometrics, identity, immunizations, health systems strengthening, health service deliver, COVID-19, COVID-19 vaccine, SARS-COV-2 vaccine, SARS-COV-2

## Abstract

The race to develop safe and effective SARS-COV-2 vaccines has moved with unprecedented speed. There are now multiple promising candidates seeking emergency use authorization from the United States Food and Drug Administration and a host of candidates positioned for approval worldwide. Attention has now turned to allocation, distribution and verification of these vaccines, yet this focus exposes that the underlying infrastructure for global delivery and monitoring is threadbare and unevenly distributed. This presents both a barrier and an opportunity to deploy sustainable infrastructure. Major global stakeholders must convene quickly, collaborate, and collectively invest in global standards, legal models, common vocabularies and interoperable biometric-supported digital health technologies. As the COVID-19 vaccine effort scales, governments, private sector and NGOs have the chance to place lasting resources needed for equitable and effective delivery that can pay dividends into the future.

As we get closer and closer to an effective SARS-CoV-2 vaccine, policymakers, academics, and scientists around the globe are turning their attention to yet another fundamental challenge - how will governments monitor and verify vaccine delivery.

An effective vaccination program will falter if we don’t invest in the information infrastructure for vaccine delivery in developed and low and middle-income countries alike. There is little evidence that we are ready for this. Glaring data gaps exist at numerous levels of global identity and health information exchange. Data on routine immunization already faces deep challenges. Studies show, for example, that despite WHO coverage estimates near 99%, up to 54% of children do not actually receive timely measles vaccinations in Bangladesh ^1^. Widespread gaps in data quality, reporting, and patient identification in routine vaccine delivery disrupt services and presage COVID-19 vaccine delivery challenges in the near future.

The supply of the first generation COVID-19 vaccines will be scarce, and each course must reach the intended recipient. Corruption, leakage, spoilage and even accidental duplications are deadly. Most current COVID-19 vaccine candidates require a two-dose course; patients will need to be reliably identified to ensure appropriate spacing of doses. Further, long-term efficacy remains to be seen and will require accurate, longitudinal patient data. Tracking patient data over time and across service delivery points requires patient identification systems. Patient identification systems will be the hardest to achieve in the places they are needed most. Many low-income countries lack a foundational government-issued ID. About one billion people lack any official civil registration.

We do have some options to face this disturbing scenario and one involves biometric digital identity.

The foundational ID challenge will not be solved in time for the release of a COVID-19 vaccine. However, organizations like Gavi have identified biometric digital identity as a potential lever to bridge the identity gap and ensure accurate data ^2^. Done properly, these systems can be privacy preserving, interoperable, portable, secure, and capable of serving both adult and children's needs. Interoperability standards will ensure these systems can plug into foundational ID programs as coverage expands over the next decade, and privacy-first architecture design is already underway in several projects ^3^.

Biometric immunization registries can deliver both COVID-19 vaccines *and* serve routine immunizations which are becoming less routine as the pandemics secondary effects become more prominent. We should be developing these architectures now - months before the ramp up of vaccine delivery begins and before further lapses in basic primary health lead to explosions of other vaccine preventable illnesses. Missing this chance could waste significant time, effort, and the chance to build forward-looking infrastructure that serves basic healthcare long into the future.

During the latest Ebola epidemic, a rush of technologies were hastily assembled to track and combat the disease, leading to massive duplication of efforts and half-built tools that were abandoned after the crisis ^4^.

We know what is coming. In the next quarter all attention will be on the allocation, distribution and verification of COVID-19 vaccine delivery. Routine immunizations will be disrupted. Investing in the infrastructure that can support COVID-19 vaccine delivery *and* routine immunizations, for everyone young and old, can ensure that we are taking advantage of this opportunity amidst the challenges and putting countries on track to fight not only this pandemic, but pressing public health needs for years to come.

Major global stakeholders must convene, collaborate, and collectively invest in global standards, legal infrastructure, common vocabularies and interoperable biometrically-supported digital health technologies. This will pay dividends long after the world’s attention has shifted. If done transparently, this infrastructure can enhance trust in vaccines, something critical to clinical trial enrolment and widespread public adoption.

We have a narrow opportunity to set the stage for fair and sustainable infrastructure across the globe. If done well, we can ensure the promise of the COVID-19 vaccine portfolio leads to future widespread vaccination - and protection - for global populations.

## Data availability

No data are associated with this article.
