# Supplementary material for: COVID-19 vaccine delivery: an opportunity to set up systems for the future
Source: Gates Open Res. 2021 Apr 14;4:182. Originally published 2020 Dec 18. [Version 2] doi: 10.12688/gatesopenres.13210.2 (PMC8173566; doi:10.12688/gatesopenres.13210.2)
Supplement: Peer review history

# Peer review: Reviewer response for version 2

**DOI:** 10.21956/gatesopenres.14475.r30576

**Published:** 2021-06-02

**Authors:** Kristin Nelson

**Affiliations:** 1 Department of Epidemiology, Rollins School of Public Health, Emory University, Atlanta, GA, USA

This version is improved. Establishing the details of how COVID-19 vaccines will be rolled out in LMIC remains a critical issue. I would still suggest that the authors provide a clear definition of what a biometric identification system would entail. (Scanning of fingerprints? Or other unique identifiers?) This is not clearly laid out and would help the reader understand what these systems would look like and the level of technology required to implement them.

Does the article adequately reference differing views and opinions?

No

Are all factual statements correct, and are statements and arguments made adequately supported by citations?

Yes

Is the Open Letter written in accessible language?

Yes

Where applicable, are recommendations and next steps explained clearly for others to follow?

Yes

Is the rationale for the Open Letter provided in sufficient detail?

Yes

Reviewer Expertise:

NA

I confirm that I have read this submission and believe that I have an appropriate level of expertise to confirm that it is of an acceptable scientific standard.

# Peer review: Reviewer response for version 2

**DOI:** 10.21956/gatesopenres.14475.r30575

**Published:** 2021-04-26

**Authors:** Alan Gelb

**Affiliations:** 1 Center for Global Development, Washington, DC, USA

I have reviewed the revised paper and it has responded to the comments.  The topic is one where we can expect a diversity of views, and the paper makes a useful contribution.

Does the article adequately reference differing views and opinions?

Partly

Are all factual statements correct, and are statements and arguments made adequately supported by citations?

Yes

Is the Open Letter written in accessible language?

Yes

Where applicable, are recommendations and next steps explained clearly for others to follow?

Partly

Is the rationale for the Open Letter provided in sufficient detail?

Yes

Reviewer Expertise:

ID systems and economic development

I confirm that I have read this submission and believe that I have an appropriate level of expertise to confirm that it is of an acceptable scientific standard.

# Peer review: Reviewer response for version 1

**DOI:** 10.21956/gatesopenres.14427.r30148

**Published:** 2021-01-20

**Authors:** Alan Gelb

**Affiliations:** 1 Center for Global Development, Washington, DC, USA

The paper addresses an important issue: the pace of Covid-19 vaccine deployment is outstripping the data systems needed to manage and document it. There is a risk of ending up with multiple, disconnected systems, many of low quality, especially in countries that do not have a strong and widely held national ID credential. Accurate identification of those receiving vaccines (and also of those administering them) is an essential part of such systems. 

 My first suggestion is to: distinguish more clearly between the stages -- national vaccine supply chain management, national vaccination rollout management, and international certification.  The priorities for ID are different across these stages;

 A second suggestion is to be clear on the role of inter-operability and standards for each stage. Within countries, it is important to head off the growth of multiple, non-interoperable, systems; therefore some national standards will be needed, including for identification. A  successful "global vaccination passport" will require the capability for mutual recognition, but will almost surely have to be built on the basis of differing national systems, just as the current passport system is built on very different registration and ID systems. 

 The third suggestion is to flag some major tradeoffs that will need to be negotiated.  One concerns the time and access to technology needed to enroll people for a vaccination program in a way that would be interoperable with a national ID. Mass vaccination needs to be fast and inclusive. It can be a driver for increasing registration, but only if this is managed well and if there is trust in the system. Another is the potential tradeoff between digital vaccination systems and privacy. Technical approaches are being proposed to give people control over their data (see the article in The Guardian, January 15) but the big concern is the credibility of many governments.  It may be that privacy and surveillance concerns slow the emergence of an international consensus on national registration systems. Nevertheless, countries will need to move forward in developing effective national vaccination management programs.  .

Does the article adequately reference differing views and opinions?

Partly

Are all factual statements correct, and are statements and arguments made adequately supported by citations?

Yes

Is the Open Letter written in accessible language?

Yes

Where applicable, are recommendations and next steps explained clearly for others to follow?

Partly

Is the rationale for the Open Letter provided in sufficient detail?

Yes

Reviewer Expertise:

ID systems and economic development.

I confirm that I have read this submission and believe that I have an appropriate level of expertise to confirm that it is of an acceptable scientific standard, however I have significant reservations, as outlined above.

# Peer review: Reviewer response for version 1

**DOI:** 10.21956/gatesopenres.14427.r30150

**Published:** 2021-01-15

**Authors:** Kristin Nelson

**Affiliations:** 1 Department of Epidemiology, Rollins School of Public Health, Emory University, Atlanta, GA, USA

This is a timely article that describes a key barrier to successful SARS-CoV-2 vaccine rollout globally – the lack of effective systems to record vaccinations. The authors propose the use of biometric digital identifiers to identify vaccinated individuals and monitor progress of vaccine administration. The rationale is clearly laid out, but several comments to improve the letter are below.

 Major comments:

 The authors recommend the use of biometric immunization registries, but do not clearly explain what they are and how they would work. A description of this system for the uninitiated reader is necessary.

 Can the authors comment on the feasibility of implementing biometric ID programs on national level (would they be implemented on a national level?) with only several months' lead time? Are projects to implement these systems already underway that could be accelerated? The authors make the case that there is a problem that needs to be solved, but not that these tools are well-positioned to solve it in the short time required.

 It may be relevant to include how these concerns are magnified given that a vaccine for COVID-19 will likely be targeted towards adults and many immunization programs in LMIC are set up primarily to administer childhood vaccinations. There is a significant disconnect here between the current structure and function of national immunization programs and what they would asked to do to widely administer a COVID-19 vaccine.

 Minor comments:

 The abstract and first paragraph should be updated to reflect the recent approval and rollout of several vaccines in high-income countries. 

 Are there alternatives to these systems for the ID challenge? If so, please briefly mention and explain why biometric identifiers are a better solution.

Does the article adequately reference differing views and opinions?

No

Are all factual statements correct, and are statements and arguments made adequately supported by citations?

Yes

Is the Open Letter written in accessible language?

Yes

Where applicable, are recommendations and next steps explained clearly for others to follow?

Yes

Is the rationale for the Open Letter provided in sufficient detail?

Yes

Reviewer Expertise:

infectious disease epidemiology, respiratory infections, vaccines

I confirm that I have read this submission and believe that I have an appropriate level of expertise to confirm that it is of an acceptable scientific standard, however I have significant reservations, as outlined above.
